# Global Research Trends and Recent Advances in Medicinal Plant-Synthesized Nanoparticles for Cancer Treatment

**DOI:** 10.3390/plants13202836

**Published:** 2024-10-10

**Authors:** Tomi Lois Adetunji, Chijioke Olisah, Marvellous Amarachi Acho, Funsho Oyetunde-Joshua, Stephen O. Amoo

**Affiliations:** 1 Agricultural Research Council—Vegetables, Industrial and Medicinal Plants, Private Bag X293, Pretoria 0001, South Africa; adetunjit@arc.agric.za; 2 Unit for Environmental Sciences and Management (UESM), Faculty of Natural and Agricultural Sciences, North-West University, Potchefstroom 2520, South Africa; 3 Institute for Coastal and Marine Research (CMR), Nelson Mandela University, P.O. Box 77000, Gqeberha 6031, South Africa; chijioke.olisah@mandela.ac.za; 4 Department of Biochemistry, Landmark University, Omu-Aran 251103, Kwara State, Nigeria; acho.marvellous@lmu.edu.ng; 5 Center of Excellence for Pharmaceutical Sciences, North-West University, Potchefstroom 2520, South Africa; 52340090@mynwu.ac.za

**Keywords:** anticancer activity, apoptosis, bibliometric analysis, drug delivery, nanomedicine, medicinal plants

## Abstract

Worldwide, cancer ranks among the foremost contributors to mortality despite recent medical progress. Alternative approaches in controlling various forms of cancer are being highly explored by researchers. This study provides the global research trends in the utilization of medicinal plant-synthesized nanoparticles for cancer treatment over the span of 18 years using scientometric analysis. Recent research advances on medicinal plant-derived nanoparticles for cancer treatment and their possible mechanisms of action were described. Relevant articles published between 2005 and 2023 were retrieved from Scopus and Web of Science and analyzed using RStudio and VOSViewer. Scientometric indicators were employed to analyze the results. The initial search returned 5695 articles, with a publication growth rate of 3.71% annually. Countries from Asia contributed the most (61.37%) to the total number of publications. The therapeutic effects of nanoparticles derived from medicinal plants can be attributed to various mechanistic pathways, including induced apoptosis from reactive oxygen species generation, as well as mitochondrial and cell membrane disruption, amongst others. Although some reported studies demonstrated promising safety and efficacy against certain cancer cells in vivo and in vitro, the little to no clinical data on medicinal plant-synthesized nanoparticles hinder the ability to make informed decisions about their clinical potential in cancer treatment.

## 1. Introduction

The utilization of plants for healing purposes dates to several centuries ago, with evidence suggesting that humans have relied on plants for treating different ailments including infection, inflammation, and colds, among others. Traditional knowledge passed down through generations has fueled explorations in this field, leading to the discovery of numerous therapeutics that have transformed healthcare. From 1940 to 2010, the United States Food and Drug Administration (FDA) approved 84 out of 173 small molecules for treating cancer, which originated from natural products and their bio-derived compounds [1]. Advancements in organic chemistry and analytical techniques have facilitated the extraction, purification, and identification of the bioactive components within plants that are responsible for their pharmacological effects. Notable examples include vincristine and vinblastine from *Catharanthus roseus*, codeine and morphine from *Papaver somniferum*, artemisinin from *Artemisia annua*, quinine from *Cinchona officinalis*, cocaine from *Erythroxylum coca*, paclitaxel from *Taxus brevifolia*, and digitoxin from *Digitalis purpurea* and *Digitalis lanata* [2]. Moreover, it is noteworthy that 25% of the drugs available in the market trace their origins back to plants [3]. These compounds, initially produced by plants for defense, pollination, and adaptation, have gained significance in drug discovery for treating several human illnesses, including cancer. 

Cancer, a non-communicable disease, is a significant public health issue due to low survival rates, disease recurrence, drug resistance, toxicity, and the non-specificity of available drugs, resulting in approximately 10 million deaths in 2020 and a projected threefold increase by 2040 [4]. Defined as abnormal cell growth with metastatic potential, cancer involves uncontrollable cell division mediated by protein upregulation and deoxyribonucleic acid (DNA) mutations, presenting a formidable treatment hurdle [5]. While chemotherapy remains the primary cancer treatment, concerns persist regarding drug toxicity, resistance from efflux transporters, and other issues, including the lower solubility and absorption, low bioavailability, rapid elimination, lower concentration at the site of action, and undesirable side effects associated with conventional drugs [6]. Addressing these challenges requires an urgent exploration of new strategies and therapies to enhance the overall survival rate and prognosis for cancer patients. Secondary plant metabolites have demonstrated crucial roles in the development of many conventional drugs currently available in the market. These metabolites, including alkaloids, terpenes, flavonoids, sterols, anthocyanins, and phenolics, among others, offer molecules with diverse structures that can serve as leads for drug discovery. Notable drugs of plant origin approved for cancer therapy include paclitaxel (Taxol^®^) and docetaxel (Taxotere^®^) for breast cancer treatment, as well as vincristine (Oncovin^®^) and vinblastine (Velban^®^) for treating various forms of cancer [7]. Additionally, the understanding of disease biology has identified druggable targets, with targeted therapy emerging as a promising approach to mitigate drug toxicity and adverse effects on healthy cells. The leaky vascular structure of cancer cells hinders successful drug delivery to the target cell or target site, prompting the use of nanoparticles (NPs) for drug delivery. 

Nanotechnology, an emerging multidisciplinary field in drug development, focuses on tiny particles in the nano range, from 0.1 to 500 nm. In cancer research, nanotechnology holds promise as an advanced system for drug delivery, diagnosis, and treatment for cancer, as well as for repairing damaged tissues and cells [8]. Nanotechnology application in medical care, known as nanomedicine, shows potential to enhance human health and well-being through precise diagnosis and targeted therapy [9]. Furthermore, the evolving focus from discovering new therapies to refining existing ones underscores the importance of incorporating nanotechnology in cancer diagnosis and treatment. Nanoparticles in cancer treatment address issues such as drug solubility and stability, protect against enzymatic degradation, enhance targeting, delivery, and distribution, facilitate controlled drug release, and reduce drug resistance [10]. Various techniques are available for synthesizing nanoparticles, including sol-gel, laser ablation, hydrothermal, microwave-assisted, and ball milling [11]. However, conventional methods have drawbacks involving the use of harmful chemicals that are not environmentally friendly. The biological method of synthesizing nanoparticles, including synthesis from bacteria, fungi, algae, and plant species, on the other hand, offers greater advantages. For example, phytochemicals extracted from plants act as reducing, capping, and stabilizing agents, thereby enhancing the effectiveness of the synthesized nanoparticles. Biosynthesis of nanoparticles is environmentally benign, non-toxic, scalable, affordable, and exhibits cellular, tissue, and organ acceptability, making it an attractive method. Different techniques are employed in biosynthesizing nanoparticles using plants, either in the crude state, post-extraction with water, or by utilizing extracted phytocompounds as reducing agents for metals, such as polyphenols, carotenoids, or essential oils [12].

Biosynthesized nanoparticles in in vivo studies have demonstrated effectiveness in the management and treatment of various cancer cells [13,14,15]. Nanotechnology-based cancer treatment products like Doxil^®^ and Abraxane^®^ have become commercially available [16]. The physical and chemical characteristics of nanoparticles endow them with unique features, facilitating easy absorption into cancer cells and interactions with cell components and cell membranes. Additionally, they can be modified to serve as vehicles for small-molecule drugs, with challenges including solubility, permeability, stability, lipophilicity, enzymatic degradation, and a short half-life. The proposed mechanistic actions underlying the activities of biologically synthesized nanoparticles in cancer cells include DNA destruction, inhibition of ribonucleic acid (RNA) topoisomerase enzyme activity, induction of apoptosis through reactive oxygen species (ROS)-mediated genetic damage, and alteration of cell membrane integrity [17].

Various plant species have been utilized for synthesizing nanoparticles (NPs), with numerous studies highlighting the positive effects of phyto-synthesized NPs against diverse carcinoma cell lines. For instance, silver nanoparticles synthesized using *Zinnia elegans* ethanolic leaf extract demonstrated anticancer effects against murine melanoma, human breast cancer, human pancreatic cancer, and human cervical cancer cell lines, demonstrating utility in bio-imaging [18]. Another example is the synthesis of copper oxide nanoparticles using the leaves of *Ormocarpum cochinchinense*, which impeded the growth of the human colon carcinoma cell line in vitro (IC_50_ = 40 μg mL^−1^) [19]. Apart from using plant extracts for synthesizing NP, the surfaces of biosynthesized NPs can be functionalized with secondary metabolites for targeted delivery into cancer cells. Raghunandan et al. [20] illustrated this approach by synthesizing silver (Ag) and gold (Au) nanoparticles using extracts from guava and clove plants. Subsequently, these NPs were functionalized with a flavonoid and tested against human cervix carcinoma, human colorectal adenocarcinoma, human kidney, and human chronic myelogenous leukemia cell lines. The study revealed inhibitory effects on the proliferation of all tested carcinoma cell lines. Additionally, bio-synthesized nanoparticles have shown promising results in targeting cancer cells while minimizing toxicity to non-cancer cells. For example, in a study reported by El-Deeb et al. [21], different concentrations of *Arthospira platensis* exopolysaccharides were used to synthesize gold nanoparticles, and their efficacy was tested against breast cancer cell lines and non-cancerous cell lines. The results revealed that one of the tested concentrations showed significant cytotoxic effects against breast cancer cells, with low to no cytotoxicity against non-cancerous cells [21]. Further, the results showed higher selectivity index values for cancerous cell lines over non-cancerous cell lines, demonstrating the greater specificity of the synthesized nanoparticles towards cancer cells. In another study [22], silver nanoparticles synthesized using *Catharanthus roseus* leaf aqueous extract showed a higher selective antiproliferative effect on cervical cancer cell lines, without affecting non-cancerous ones. The enhanced selectivity was attributed to the natural compounds present in the plant extract used that can help to reduce the potential cytotoxicity risk in normal cells [22]. However, these effects may not be seen with chemically synthesized nanoparticles due to the utilization of toxic reducing agents. While many research studies support the considerable potential of medicinal plant-synthesized nanoparticles in treating/managing various types of cancer, a comprehensive scientometric analysis is beneficial to assess the progress and future projections of medicinal plant-synthesized nanoparticles for cancer treatment. Therefore, this study is aimed at investigating the advancements over an approximately 19-year period (January 2005 to October 2023) by utilizing a scientometric approach while projecting future developments.

## 2. Materials and Methods

**Figure 1:**
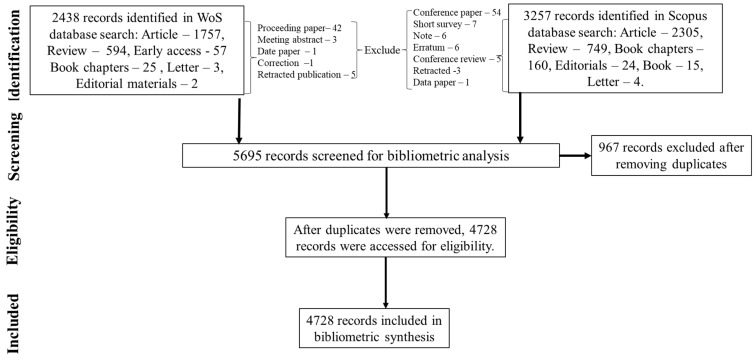
Flow chart of literature selection.

We adopted the protocol for data retrieval from Aria and Cuccurullo [23], Adetunji et al. [24], and Bayode et al. [25]. In summary, we identified studies published on the use of medicinal plants as a medium to develop nanoparticles for cancer therapy by using the following keywords on the Clarivate Web of Science (WoS) Core Collection platform: Topic Search, TS = (“plant*” OR “medicinal plant*”) AND TS = (“nanoparticle*” AND “cancer*”). This process involved gathering articles published between 1 January 2005 and 23 October 2023. We focused on specific types of publications: research articles (*n* = 1757), review articles (*n* = 594), early access materials (*n* = 57), book chapters (*n* = 25), letters (*n* = 3), and editorial content (*n* = 2). We did not include certain document types like proceeding papers (*n* = 42), meeting abstracts (*n* = 3), data papers (*n* = 1), corrections (*n* = 1), and retracted publications (*n* = 5) because these are primarily preliminary data rather than full articles. After excluding these document types, we identified a total of 2438 articles that met our search criteria (see Figure 1). Similarly, the following keywords were inputs into the Scopus database to retrieve the target article metadata: TITLE-ABS-KEY (“plant*” OR “medicinal plant*”) AND TITLE-ABS-KEY (“nanoparticle*” AND “cancer*”). We focused our search on specific document types, including research articles (*n* = 2305), review articles (*n* = 749), book chapters (*n* = 160), editorials (*n* = 24), books (*n* = 15), and letters (*n* = 4). Furthermore, some document types, including conference papers (*n* = 54), short surveys (*n* = 7), notes (*n* = 6), errata (*n* = 6), conference reviews (*n* = 5), retracted publications (*n* = 3), and data papers (*n* = 1), were excluded from the search. Overall, our search identified a total of 3257 documents (see Figure 1).

We downloaded 2438 publications from the Web of Science database and 3257 publications from the Scopus database. Subsequently, we imported them into RStudio (Version 1 April, 1106 © 2009–2021 RStudio, PBC) for bibliometric analysis. After combining the records from both databases and removing duplicates using R commands, we obtained 4728 unique documents. For document analysis, we utilized codes outlined by Aria and Cuccurullo [23] to examine bibliometric indices, including yearly publication frequency, number of articles, authors, citation metrics, and country collaborations. Additionally, we employed VOSviewer (version 1.6.15 © 2009–2022) to visually represent topic domains related to the research. The Kolmogorov–Smirnoff (K-S) *p*-value, goodness of fit, and β-coefficient from RStudio (Version 1 April, 1106 © 2009–2021 RStudio, PBC) were also applied to comprehend the yearly production frequency of publications. 

## 3. Results

### 3.1. Analysis of Annual Publications

**Figure 2:**
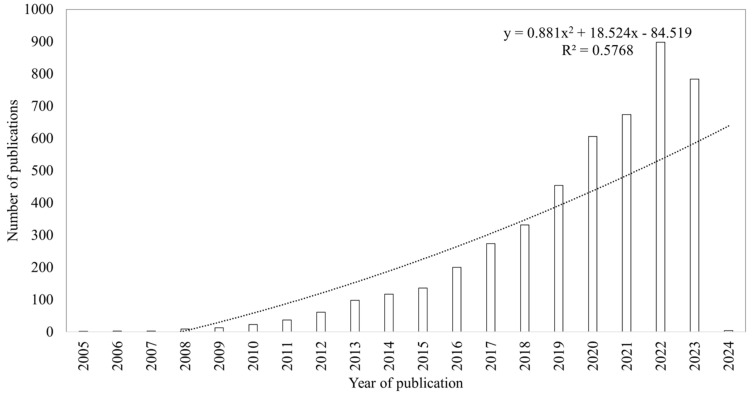
Annual scientific production on medicinal plant-synthesized nanoparticles for cancer treatment (1 January 2005–23 October 2023).

The 4728 documents were classified into six categories, predominantly research articles (*n* = 3593; 75.99%), followed by reviews (*n* = 944; 19.97%), book chapters (*n* = 150; 3.17%), editorial materials (*n* = 19; 0.40%), books (*n* = 15; 0.32%), and letters (*n* = 7; 0.15%). Originating from 1168 diverse sources (journals, books, etc.), the documents included 19,869 keywords and 8282 author’s keywords. After excluding 119 single-authored publications, the total number of authors for the published multiple-author articles amounted to 16,634, averaging 3.52 authors per document and 0.284 documents per author (Supplementary Table S1). Figure 2 illustrates the trend of the annual scientific publications on medicinal plant-synthesized nanoparticles for cancer treatment from 2005 to 2023. Notably, a consistent rise in annual scientific publications was observed, progressing from 2 articles in 2005 to 784 articles in 2023, depicting an annual growth rate of 3.71%. The peak year for publications was 2022, with 898 articles. 

The analysis, utilizing a polynomial approach, demonstrated a strong positive correlation (r^2^ value of 0.958) between the year of publication and the number of articles. Additional statistical metrics, including the Kolmogorov–Smirnoff goodness-of-fit (0.541) and the β-coefficient (2.403), further support the anticipation of a continued increase in scientific publications in this field of research in upcoming years. 

### 3.2. Contribution and Collaboration Network between Countries

**Figure 3:**
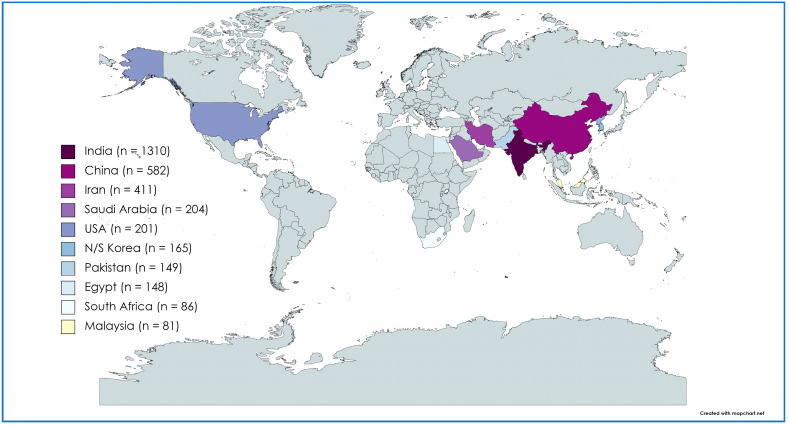
Heat map showing global distribution of the 10 foremost nations researching medicinal plant-synthesized nanoparticles for cancer treatment.

In Figure 3, the heat map reveals the global distribution of the top 10 countries contributing to research articles on medicinal plant-synthesized nanoparticles for cancer treatment. Predominantly, India, China, Iran, Saudi Arabia, and the USA stood out as significant. Among the first 50 countries analyzed, India led with 1310 publications, accounting for 27.7% of all research in this field. China ranked second with 582 publications, followed closely by Iran (411 publications), Saudi Arabia (204 publications), and the USA (201 publications). Notably, India (32,083 citations) and China (12,750 citations) were the top two countries in terms of citations, aligning with their high publication output. The USA ranked third in citations (9065), followed by Iran (8811) and North Korea and the South Korea (6362). 

**Figure 4:**
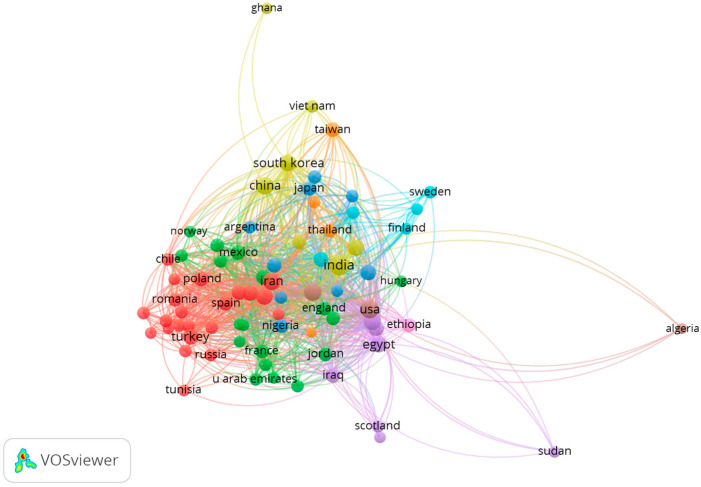
Collaboration network on medicinal plant-synthesized nanoparticles for cancer treatment research among top contributing countries.

A network visualization map was constructed for visualizing the international collaboration network in medicinal plant-synthesized nanoparticles for cancer treatment research (Figure 4). The sizes of circles correlate with the numbers of articles, connections between nodes depict citation relationships, and line thickness indicates the rates of collaboration between countries. Countries with higher productivity (measured by publication count and citation count) showed increased collaboration links. 

### 3.3. Analysis of Journals Publishing Medicinal Plant-Synthesized Nanoparticles for Cancer Treatment

In bibliometric analysis, a critical objective is to identify and assess the distribution of journals that publish relevant research. This aids researchers in identifying suitable journals for publishing their own articles. Hence, in terms of research and cited articles, mainstream journals with considerable influence in a particular research area often feature a significant number of articles. Table 1 shows the top 10 journals publishing research outputs on medicinal plant-synthesized nanoparticles for cancer treatment during the study period. The impact factor of these journals ranged from 4.6 to 8.2. The journals were published by Nature, Taylor & Francis, Elsevier (Science Direct), and the Multidisciplinary Digital Publishing Institute (MDPI). From the analysis, the International Journal of Nanomedicine stood out as the most prolific journal, publishing 121 articles, followed by Molecules (105 articles), Journal of Drug Delivery Science and Technology (102 articles), Artificial Cells Nanomedicine and Biotechnology (86 articles), Pharmaceutics (72 articles), Scientific Reports (57 articles), Nanomaterials (55 articles), International Journal of Molecular Biology (54 articles), International Journal of Biological Macromolecules (52 articles), and the Arabian Journal of Chemistry (51 articles).

Supplementary Table S2 displays the foremost 10 authors on medicinal plant-synthesized nanoparticles. A total of 16,634 authors contributed to publications on medicinal plant-synthesized nanoparticles for cancer treatment during the study period. The most prolific author, Steinmetz N., authored 74 publications, followed by Wang Y. (63 publications), Singh S. (54 publications), Khan M. (52 publications), Zhang X. (50 publications), Wang C. (45 publications), Zhang Y. (44 publications), Li Y. (41 publications), Liu Y. (41 publications), and Wang J. (39 publications). Collectively, the publications by these authors account for 10.63% of the total articles published. Regarding citations, Wang Y. emerged as the top cited author with a total citation count (TC) of 10,770, followed by Zhang H. (9238 TC), Zhang Y. (8698 TC), Zhang X. (8380 TC), Zhang Z. (7364 TC), Li M. (7003 TC), Chen Y. (6873 TC), Wang C. (6734 TC), Singh S. (6061 TC), and Kim J. (6051 TC). Notably, only 5 of the foremost 10 most published authors overlap with the foremost 10 most cited authors.

Supplementary Table S3 presents the ten most cited publications, with two articles published in Colloids and Surfaces B: Biointerfaces, while the others are distributed across various journals, including “Pharmacological Reviews”, “Saudi Pharmaceutical Journal”, “International Journal of Nanomedicine”, “Molecules”, “International Journal of Molecular Sciences”, “Biomaterials”, “International Journal of Pharmaceutical Sciences”, and “Research and Theranostics”. The number of citations for these top 10 articles ranges from 404 to 693, with total citations per year ranging from 32.2 to 101.0. Notably, five of these articles have received more than 450 citations.

### 3.4. Analysis of Keywords and Thematic Categorization

**Figure 5:**
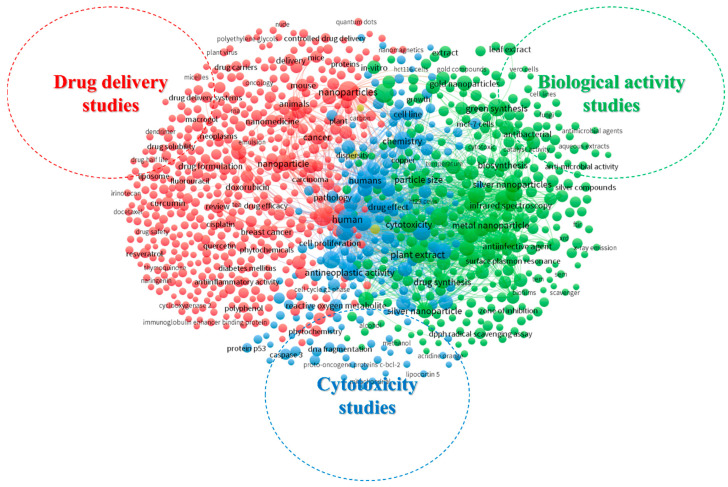
Thematic classification of articles on medicinal plant-derived nanoparticles for cancer treatment based on keyword analysis.

In the keyword co-occurrence analysis, the foremost 50 most relevant keywords (ID and DE) in medicinal plant-synthesized nanoparticles for cancer treatment publications were analyzed. These were classified into themes. The resulting thematic classification of the keywords showed three clusters (Figure 5). The first cluster (red cluster) relates to drug delivery studies of medicinal plant-synthesized nanoparticles for cancer treatment and consists of keywords such as drug carriers, drug delivery systems, drug formulation, phytochemicals, breast cancer, drug efficacy, and polyphenols, among others. The second cluster (green cluster) focused on other biological activity studies of plant-synthesized nanoparticles and consists of terms such as silver nanoparticles, DPPH radical scavenging activity, antimicrobial activities, anti-infective agent, gold nanoparticles, metal nanoparticles, and zone of inhibition. The last cluster (blue cluster) consists of terms relating to the cytotoxic effects of medicinal plant-derived nanoparticles on cancer cells. Terms such as antineoplastic activity, reactive oxygen metabolite, DNA fragmentation, cytotoxicity, and cell lines are included in this cluster.

### 3.5. Recent Research Advances in Medicinal Plant-Synthesized Nanoparticles for Cancer Treatment

Cancer is a non-communicable disease that has led to significantly increased morbidity in recent years, amounting to over 70% of premature deaths globally, and a 47% increase in deaths resulting from cancer is projected by 2040 [26,27]. Cancer ranks as the second most prevalent global cause of mortality, with more than 80% of these fatalities occurring in developing countries [27]. Recent reports indicated that a total of approximately 10 million newly diagnosed cancer cases are documented yearly, with 5.5 million of these cases recorded in less developed countries, while 4.7 million cases are recorded in developed countries [27]. The disturbing rise in the number of cancer cases has increased pressure on healthcare facilities, particularly in underdeveloped countries with high incidences of cancer and insufficient healthcare services [28].

Although knowledge of molecular biology and tumor biology has increased in recent years, aiding in cancer treatment, successful cancer treatment is still difficult to achieve. The failure of these treatment strategies often stems from the ability of diverse cancers to acquire mutations over time, rendering them resistant to treatment [29,30]. In the past few years, the treatment options available to cancer patients are radiotherapy and surgery, commonly used for the treatment of solid localized tumors, as well as chemotherapy, employed for the treatment of hematologic cancers and metastatic solid neoplasms. These treatment options are usually used singly or combined [31]. These treatment options are often linked to a variety of side effects such as severe toxicity and the occurrence of secondary tumors. Other drawbacks of these treatment options include non-selectivity and the development of poly-drug resistance [6]. Furthermore, cancer patients experience difficulties in handling the cost of treatment [32,33]. 

The exploration of novel treatment strategies in managing and/or treating cancer has resulted in heightened interest in nanoparticles in recent years. Nanotechnology stands as one of the contemporary scientific breakthroughs fueling industries with public and financial interests. Nanoparticles possess better or significantly improved physical, chemical, and electrical properties such as morphology, size, and distribution. These properties are useful in the beauty, food, agriculture, manufacturing, packaging, and drug-manufacturing industries [34,35]. Nanotechnology has been used in the past few years as a safer and more effective means for cancer diagnosis and treatment. The ideal nanoparticles for use in cancer treatment are those with sizes ranging between 10 and 100 nm. This is because they possess good pharmacokinetics, improved permeability and retention effects, and are more effective in drug delivery [36,37]. Nanoparticle-based cancer treatment strategies have been reported to demonstrate precise targeting, few side effects, and drug sensitizing effects [38]. Particularly, scientists have discovered that biologically synthesized nanoparticles exhibit superior therapeutic properties compared to nanoparticles obtained through other physicochemical means [37]. Phyto-synthesized nanoparticles offer advantages over other biological methods involving fungi, bacteria including actinomycetes, and algae [37]. 

Further, phyto-synthesized nanoparticles offer several advantages compared to chemically synthesized nanoparticles. They are typically solvent-free, less toxic, eco-friendly, cost-effective, simple to produce, easily stable, and monodispersed [38,39]. Another main advantage of plant-synthesized nanoparticles is that different plant parts, including roots, leaves, fruits, peels, and stems, among others, may be utilized in the process of synthesis [40]. The therapeutic activities of nanoparticles synthesized from medicinal plants have been ascribed to the occurrence of various bioactive metabolites with therapeutic activities. These compounds include phenolics, terpenoids, saponins, amino acids, polysaccharides, enzymes, and vitamins [40]. This section highlights some recent research advances in medicinal plant-synthesized nanoparticles for cancer treatment and the probable mechanism of their anticancer properties.

#### 3.5.1. Anticancer Activities of Medicinal Plant-Synthesized Nanoparticles

The anticancer potential of medicinal plant-synthesized nanoparticles has been reported in various studies. The physical properties and anticancer effects of medicinal plant-synthesized nanoparticles are summarized in Table 2 below. Several medicinal plant-synthesized nanoparticles (MPSNPs) have been documented to exhibit cytotoxic effects against diverse cancer types. Reports of cytotoxicity of MPSNPs against breast carcinoma cells [41], human colon carcinoma cell lines [42], human bladder cancer cells [43], lung cancer [44], cervical cancer [45], and prostate cancer [46] are available. 

##### Breast Cancer

Erdogan et al. [47] asserted that silver nanoparticles synthesized from *Cynara scolymus* leaf extracts possess anticancer properties. The capability of the nanoparticles to induce apoptosis in MCF-7 breast carcinoma cells was investigated via intracellular ROS analysis, Bax/Bcl-2 analysis, antioxidant enzyme levels, and Hoechst staining. The results revealed that the synthesized nanoparticles caused mitochondrial damage as well as increased intracellular ROS production. A significant reduction in cell migration, Bcl-2 suppression, and the upregulation of Bax expression were also observed. The mechanism of the anticancer effects of the nanoparticles was promoting ROS generation through the modulation of the induction of mitochondrial apoptosis in MCF-7 breast carcinoma cells.

Silver nanoparticles synthesized from *Mangifera indica* ethanolic bark extract were assessed for their anticancer activities. The nanoparticles were reported to possess strong anticancer activities against the Michigan Cancer Foundation-7 breast cancer cell line, with 80.1% cytotoxicity at 50 μg mL^−1^. The authors concluded that the synthesized silver nanoparticles from *M. indica* bark can be used as anticancer agents [35]. Also, the biosynthesis of silver nanoparticles using Copperpod plant aqueous leaf extracts were studied for their anticancer potential on HepG2, MCF-7, and A549 cancerous cells. The results indicated the half maximal inhibitory concentration values of the nanoparticles against the HepG2, MCF-7, and A549 cancer cells were 69 μg/mL, 62 μg/mL, and 53 μg/mL, respectively. The results of the study showed that Copperpod plant-synthesized silver nanoparticles demonstrated significant anticancer activities against the carcinoma cell lines [48]. Chavan et al. [49] investigated the anticancer activities of phyto-synthesized iron and silver nanoparticles using *Blumea eriantha* whole plant alcoholic extract against MCF-7 breast carcinoma cell lines. In comparison with the control, the nanoparticles exhibited significant anticancer activity via induced apoptosis in the MCF-7 cells. The authors concluded that nanoparticles synthesized from *B. eriantha* could potentially pave the way for novel avenues in anticancer therapies. 

Silver nanoparticles were synthesized from garlic cloves and their anticancer effect was evaluated against breast cancer [41]. The synthesized nanoparticles exhibited notable anticancer properties, significantly reducing the viability of MCF-7 at 100 μg mL^−1^ after 24 h. An observable change in the morphology of the cancer cells was also detected. Furthermore, the nanoparticles demonstrated no toxicity towards human HEK293 embryonic cells, suggesting their safety. In a different study by Unuofin et al. [50], using *Vernonia mespilifolia* extract, silver-platinum bimetallic bio-synthesized nanoalloys were investigated for their cytotoxic effect against MCF-7. The phyto-synthesized nanoparticles hindered the proliferation of the carcinoma cells. The nanoparticles demonstrated a selective cytotoxic effect towards MCF-7, as they were comparatively non-toxic to the HEK 293 normal cell line. 

##### Liver Cancer

Javid and Dilshad [33] evaluated the anticancer activity of bio-synthesized silver nanoparticles using *Artemisia carvifolia* extract against liver cancer, with a target on the Rap2A gene. The results showed significant cytotoxicity against HepG2 cancer cell lines, with an IC_50_ value of 2.57 μM for silver nanoparticles. The Rap2 gene was also suppressed in the nanoparticle-treated cells. The apoptotic effect of the nanoparticles was further examined by assessing the levels of expression of apoptotic pathway genes and proteins. The results revealed increased expression of these genes as well as increased protein levels. Hemlata et al. [51] evaluated the antiproliferative activity of green-synthesized silver nanoparticles using *Cucumis prophetarum* aqueous leaf extract, assessed by a 3-(4,5-dimethylthiazol-2-yl)-2,5-diphenyltetrazolium bromide (MTT) assay on HepG2 cells. The nanoparticles were potent against the liver cancer cells by inducing a decrease in viability of the cells, with an IC_50_ value of 94.2 μg/mL. 

##### Cervical Cancer

Al-Sheddi et al. [45] investigated the anticancer potential of silver nanoparticles synthesized from the aqueous extract of the aerial parts of *Nepeta deflersiana* against human cervical cancer (HeLa) cells. Results revealed that the nanoparticles induced SubG1 arrest and apoptotic/necrotic cell death in the HeLa cells. This suggests the anticancer potential of silver nanoparticles synthesized from *Nepeta deflersiana* against cervical cancer cells. As reported by Rajasekharreddy and Rani [52], silver nanoparticles derived from the seed extract of *Sterculia foetida* were examined on cervical cancer cell lines (HeLa). The study found that the nanoparticles significantly suppressed the multiplication of HeLa cells at a dose of 16 g/mL.

Silver nanoparticles obtained from *Punica granatum* aqueous leaf extract exhibited anticancer activity effects on human cervical carcinoma cells (HeLa) [53]. The anticancer investigation of the synthesized nanoparticles was carried out using the MTT, DNA fragmentation, and lactate dehydrogenase assays. The determination of quantity of LDH release can facilitate the investigation of the toxic effect of nanoparticles on cancer cells. The results indicated that the nanoparticles hindered cell proliferation and caused cell senescence in the HeLa cells in a concentration-dependent manner. The results from the DNA fragmentation assay showed that the nanoparticles induced apoptosis by cleaving the nuclear DNA of HeLa cells. The study concluded that the nanoparticles synthesized from the aqueous leaf extract of pomegranate could be useful in drug delivery and can be used as a therapeutic agent for cancer treatment [53].

##### Lung Cancer

In a study by Alharbi and Alsubhi [44], the cytotoxic effect of synthesized silver nanoparticles from *Azadirachta indica* fruit and leaf extracts evaluated against H1975 cell lines showed high toxicity against the lung cancer cells. The authors noted that the nanoparticles’ potent anticancer activity may stem from their capacity to induce apoptosis, activating ROS within cancer cells and leading to cell membrane degradation, oxidative stress, and apoptosis. They suggest further investigations to assess the toxicity of nanoparticles on normal healthy cells.

In a study by Sre et al. [54], silver nanoparticles synthesized from *Erythrina indica* demonstrated cytotoxic effects on breast and lung cancer cell lines. The researchers concluded that the nanoparticles, ranging from 20 to 118 nm, exhibited significant cytotoxicity against MCF-7 and HEP G2 cancer cells, suggesting their potential as promising chemopreventive agents. As reported by Lakshmanan et al. [55], silver nanoparticles synthesized from *Cleome viscosa* fruit extract were analyzed for their anticancer activities. The synthesized nanoparticles showed strong anticancer activity against lung (A549) and ovarian (PA1) cancer cell lines, with the lowest IC_50_ concentrations at 28 and 30 μg/mL, respectively [55]. He et al. [56] demonstrated the antitumor activity of silver nanoparticles synthesized using the peel of *Dimocarpus longan* against lung cancer using in vitro and in vivo assays. The cytotoxic impact of the synthesized nanoparticles was investigated on human lung cancer H1299 cells in vitro using MTT and trypan blue assays. Apoptosis was assessed through morphological analysis, while the expressions of phosphorylated STAT3, Bcl-2, survivin, and caspase-3 were also evaluated. The results revealed that in the H1299 cells, the synthesized nanoparticles displayed concentration-dependent cytotoxicity and stimulation of apoptosis, decreased Bcl-2, and increased expression levels of caspase-3 and survivin. The nanoparticles also significantly suppressed H1299 tumor growth in a xenograft severe combined immunodeficient (SCID) mouse model. The results demonstrated the anticancer activities of the nanoparticles and suggest their application in lung cancer treatment.

##### Prostate Cancer

The antiproliferative effect of silver nanoparticles synthesized using *Alternanthera sessilis* aqueous leaf extract demonstrated increased anticancer activity against prostate cancer (PC-3) cells in a dose-dependent manner [57]. The nanoparticles also induced a morphological change in the cells, as cancer cell membrane lysis was observed after 48 h. A reduction in the number of the PC-3 cells was also observed at 12.5 and 25 μL/mL of nanoparticles. In a separate study, the anticancer activity of silver nanoparticles synthesized using *Dimocarpus longan* peel aqueous extract was investigated against prostate cancer. The cytotoxicity effect of the nanoparticles was determined using the human prostate cancer PC-3 cells in vitro, assessed by the trypan blue assay. The expressions of phosphorylated STAT3, Bcl-2, survivin, and caspase-3 were also determined. The results showed that the nanoparticles induced a concentration-dependent cytotoxicity against the PC-3 cells. The nanoparticles also induced the downregulation of STAT3, survivin, and Bcl-2, as well as the upregulation of caspase-3. The findings in this study showed a potential anticancer application of the nanoparticles for prostate cancer treatment [56].

Silver nanoparticles synthesized with *Carica papaya* leaf extract was reported to induce cell cycle arrest and apoptosis in human prostate (DU145) cancer cells [58]. The cytotoxic effect of the nanoparticles was investigated against the prostate cancer cells and non-tumorigenic human keratinocyte cells. According to the results, the nanoparticles induced a decrease in cell viability by 74% in DU145 cells, and were relatively less toxic to the normal cells, as no significant decrease in cell viability of the normal cells was observed.

##### Colon Cancer

Fadaka et al. [42] investigated the cytotoxicity of nanoparticles synthesized with gum arabic against colon cancer cells. They employed the MTT assay to assess the cytotoxicity of the nanoparticles on two colon cancer cell lines (Caco-2 and HT-29), as well as non-cancerous cells (KMST-6). The results revealed significant cytotoxic effects of the nanoparticles on the cancer cells at concentrations ≥50 μg/mL, while normal cells were also affected at the same concentration. The study concluded that the nanoparticles exhibited non-selective toxicity, impacting both cancer and non-cancer cells, with less than 15% viable cells observed across all concentrations.

#### 3.5.2. Possible Mechanism of Action of Anticancer Activities of Medicinal Plant-Synthesized Nanoparticles

**Figure 6:**
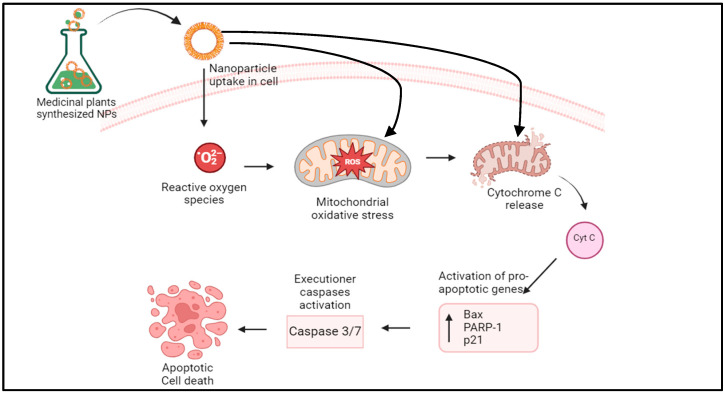
Possible mechanism of action of the anticancer activities of medicinal plant-synthesized nanoparticles. The figure illustrates the mechanistic pathways underlying the anticancer effects of medicinal plant-synthesized nanoparticles, including reactive oxygen species (ROS)-mediated apoptosis, mitochondrial and cell membrane disruption, and upregulation of apoptotic genes.

MPSNPs may bring about their anticancer activity by interacting with biomolecules, inducing several physiological effects such as oxidative stress, interaction of the nanoparticles with cellular proteins and DNA, increased membrane permeability, mutations, signaling pathway activation, and conformational changes leading to cell lysis [39]. According to several studies, the cell-killing effects of MPSNPs are attributed to the reactive oxygen species (ROS)-mediated toxicity. Cancer cells are highly susceptible to electron transfer from MPSNPs, resulting in cellular and metabolic changes and thereby destroying the cancerous cells [43,59]. ROS generation, including superoxide radicals and hydrogen peroxide, can result in the alteration of the mitochondrial transmembrane, leading to uncoupling of the respiratory mechanisms and eventually, oxidative stress. MPSNPs can alter signal transduction pathways, thereby causing the death of cells through apoptosis. This occurs through the translocation of Bax into the mitochondria and the release of cytochrome C into the cytosol, coupled with cell cycle cessation during the growth and DNA synthesis phases (Figure 6). Additionally, MPSNPs can upregulate apoptotic genes including Bax, p53, p21, and poly-ADP ribose polymerase-1, facilitating cell death [59].

## 4. Discussion

The intersection of medicinal plants and nanotechnology has introduced new possibilities in cancer therapy [60]. Nanoparticles synthesized using medicinal plants are gaining increasing attention due to their potential in delivering therapeutic agents with enhanced efficacy and reduced side effects [61]. This upward trajectory is evident in the rising number of annual scientific articles pertaining to the focus of this study, as shown in Figure 2, reflecting the sustained interest of researchers in this field over the past decade. The results revealed a consistent rise in cumulative research articles on medicinal plant-synthesized nanoparticles for cancer treatment over the last 19 years. Notably, there was a significant increase in scientific publications from 2017 to 2023, highlighting the growing interest in this field. The slight deviation from the expected growth trend in the year 2023 may be attributed to our partial year analysis (up to 23 October 2023), whereas full-year data and future research will most likely maintain an upward trajectory, driven by increasing interest in cancer treatment applications. Most of the published articles were research papers, with fewer being review articles. The expectation is that the number of research articles will continue to grow, reflecting an upward trend in the years ahead as this field evolves. This upward trajectory may be closely linked to the global increase in the cancer burden, with a projected 77% rise by 2050 associated with population growth and ageing, and exacerbated by exposure to risk factors including alcohol, tobacco, obesity, and air pollution [62]. 

The results depicted in Figure 3 showed that the majority of research articles on medicinal plant-synthesized nanoparticles for cancer treatment originated from Asian countries. The high publication and citation output from countries originating in Asia may be attributed to the region’s significant contribution to global cancer incidence (49.3%), mortality (58.3%), and 5-year prevalence (40.8%), and thereby the prioritization of cancer research [63]. Furthermore, the use of phytomedicine has been a popular source of healthcare in Asian countries. India and China lead in plant-synthesized nanoparticle research, driven by their rich traditions of plant-based medicine, abundant diversity, government initiatives, and cultural acceptance. In India, over 20,000 medicinal plant species are recorded. Similarly, approximately 400 known plant species are used in Chinese herbal medicine. Notably, 45% of the world’s regions currently utilize Chinese herbal medicine (CHM) in healthcare, with the global CHM trade reaching $40 billion annually [64]. This unique blend of factors has enabled a thriving research environment, driving innovations in cancer treatment research using plant-synthesized nanoparticles and contributing the most to global research outputs in this field. Similarly, articles authored by researchers from Asian countries garnered the highest citation count, and authors from these countries maintain the closest collaborative networks with other countries. Notably, India demonstrated close collaborations with countries including China, Iran, the USA, and South and North Korea, underscoring its crucial role in this research field (Figure 4). Additionally, the rich biodiversity and traditional knowledge in India, and the global demand for affordable healthcare, standardization of herbal medicines, and advancements in nanotechnology and delivery systems may have fostered its research collaborations with these countries. The top 10 authors (Supplementary Table S2) in this study remarkably align with the top publishing countries (Figure 3). It is expected that countries with top authors are likely to lead in terms of research output, expertise, and innovation.

The analysis of the top ten journals (Table 1) publishing research on medicinal plant-synthesized nanomaterials for cancer treatment showed they were notable publishers including Nature, Taylor & Francis, Elsevier (ScienceDirect), and the Multidisciplinary Digital Publishing Institute (MDPI). The analysis of these journals revealed articles grouping into three main subject areas: nanomedicine, drug delivery, and nanotechnology. These thematic research areas for medicinal plant-synthesized nanoparticles play a crucial role in advancing healthcare and offering targeted and efficient treatment strategies, especially in cancer therapy, drug delivery, and personalized medicine. The published research reflects continuous efforts to expand scientific understanding and technological innovation in this field.

The top 10 most cited articles covered various aspects such as biosynthesis and characterization, biological and pharmacological activities, drug delivery, bioactive metabolite profiling, and mechanisms of action in medicinal plant-synthesized nanoparticles for cancer treatment. These thematic research foci are important because they not only contribute to the scientific understanding of nanotechnology and cancer biology, but also hold great therapeutic potential for cancer treatment. For example, the most cited paper, titled “Biosynthesis of metallic nanoparticles using plant derivatives and their new avenues in pharmacological applications—An updated report”, is a review that discusses the potential therapeutic applications of biosynthesized nanomaterials from medicinal plants in controlling and treating various diseases, including HIV, malaria, hepatitis, cancer, and other acute diseases. The article emphasizes the abundance of natural compounds in plants, including saponins, tannins, alkaloids, steroids, flavonoids, and other nutritional compounds, which serve as stabilizing and reducing agents in the bioreduction reaction during the synthesis of novel metallic and other nanoparticles [65]. Furthermore, the second most cited article discusses enhancing the formulation and delivery system of curcumin to boost its anticancer activities. One such suggested approach to increase curcumin’s bioavailability in cancer therapy is through nanocurcumin—specifically, polymeric nanoparticle-encapsulated curcumin. The article also reports the anticancer efficacy of this formulation in in vivo models [66]. 

The keyword analysis in Figure 5 indicates that medicinal plant-synthesized nanoparticles for cancer treatment are primarily associated with pharmacy, plant science, chemistry, biochemistry, toxicology, and medicine. These studies primarily focus on drug delivery effects, cytotoxicity studies, and other biological activities that contribute to the efficacy of nanoparticles derived from medicinal plants in treating cancer. Overall, the global research trend in this field involves an increasing focus on identifying novel medicinal plants for nanoparticle synthesis. Collaborative efforts among researchers aim to standardize green synthesis methods, incorporating interdisciplinary approaches that span nanotechnology, pharmacology, and botany.

Although medicinal plants have been used in nanoparticle biosynthesis with other metals like zinc, gold, copper, silver, and iron for diverse applications in healthcare, food, agriculture, and medicine [67], silver nanoparticles synthesized from medicinal plants have primarily been studied for their potential applicability in the diagnosis and treatment of cancer, owing to their distinctive physical and chemical characteristics with minimal systemic toxicity [68,69]. Recent research on the anticancer activities of nanoparticles synthesized from medicinal plants highlights these plants as abundant sources of bioactive compounds, with many plant extracts serving as both reducing and stabilizing agents [65,70]. The green synthesis approach provides environmentally friendly and economical alternatives to conventional methods. Different medicinal plants contribute to the biosynthesis of diverse nanoparticles, including gold, silver, zinc oxide, and iron oxide, each exhibiting unique properties for diverse cancer treatments. 

The multifunctional biological properties of medicinal plants, such as their antioxidant effects leading to apoptosis, cytotoxic effects, and others, contribute significantly to the therapeutic effect of medicinal plant-synthesized nanoparticles [69]. Further, bioactive molecules in medicinal plant extracts contribute to their targeted effects on cancer cells, enhanced drug delivery, imaging, and therapeutic outcomes. The efficacy of medicinal plant-synthesized nanoparticles in inhibiting cancer cells is attributed to their capability to target various signaling pathways implicated in cancer development and induce apoptosis. These nanoparticles can interfere with cell proliferation, angiogenesis, metastasis, and drug resistance mechanisms, thereby exerting synergistic effects against cancer cells [39]. Furthermore, they can enhance the efficacy of conventional chemotherapeutic agents by overcoming drug resistance and improving their bioavailability. These nanoparticles also exhibit selective cytotoxicity towards cancerous cells without affecting normal cells, which is crucial for minimizing side effects in cancer treatment [41].

## 5. Conclusions and Future Perspectives

This study employed scientometric analysis to provide valuable insights in the field of medicinal plant-synthesized nanoparticles for cancer treatment, highlighting research trends, recent advances, and areas for future exploration and development. The global increase in research contributions to the field of medicinal plant-synthesized nanoparticles holds promise for enhancing cancer treatment outcomes. However, addressing some specific research gaps is crucial. Firstly, uniform protocols for nanoparticle synthesis from medicinal plants are needed to facilitate comparative studies, ensure accurate result interpretation, and establish best practices. Standardization can also foster collaboration among researchers, which can help in the translation of nanoparticles from the laboratory to industrial-scale production.

While there is a general belief in the safety of medicinal plants and their extracts, it is essential to acknowledge the existence of toxic medicinal plants when not used at the correct dosage. Consequently, comprehensive toxicity studies are imperative to evaluate the safety of medicinal plant-synthesized nanoparticles for therapeutic use. While many studies have focused on the potency of medicinal plant-synthesized nanoparticles, less attention has been given to their selectivity and safety on non-targeted cells. Long-term toxicity studies are necessary to comprehend the cumulative effects of prolonged exposure and investigate potential chronic toxicity, assessing the persistence of nanoparticles in the body over time. Given the promising results of medicinal plant-synthesized nanoparticles with metals such as zinc, gold, iron, and copper in various agricultural, food, cosmetic, and consumer goods applications, more studies to explore their efficacy in cancer therapy is warranted. Finally, despite recent research advances primarily consisting of in vitro studies, it is important to conduct more in vivo investigations and consider transitioning reasonably to clinical trials. This approach is crucial for making well-informed decisions about the clinical potential of medicinal plant-synthesized nanoparticles in the fight against cancer. 

## Figures and Tables

**Table 1:** Top 10 productive journals and their impact factors (IFs) publishing articles on medicinal plant-synthesized nanoparticles for cancer treatment from 2005 to 2023.

S/N	Journal Name	Number of Articles	2022 Impact Factor
1	International Journal of Nanomedicine	121	8.0
2	Molecules	105	4.6
3	Journal of Drug Delivery Science and Technology	102	5.0
4	Artificial Cells Nanomedicine and Biotechnology	86	5.8
5	Pharmaceutics	72	5.4
6	Scientific Reports	57	4.6
7	Nanomaterials	55	5.3
8	International Journal of Molecular Biology	54	5.6
9	International Journal of Biological Macromolecules	52	8.2
10	Arabian Journal of Chemistry	51	6

**Table 2:** Medicinal plant-synthesized nanoparticle characteristics and anticancer efficacy.

Type of Nanoparticles (NPs)	Plant Species	Plant Part	NPs Particle Size	Type/Shape of NPs	Cell Lines	Effect	References
Silver nanoparticles (AgNps)	*Cynara scolymus*	Leaf	98.47 ± 2.04 nm	Spherical	Breast cancer (MCF-7)	AgNps from *C. scolymus* leaf extract demonstrated efficient anticancer activities via mitochondrial apoptosis in MCF-7 cells.	[47]
AgNps	*Mangifera indica*	Bark	56–89 nm	Spherical	MCF-7	AgNps from *M. indica* bark extract showed cytotoxicity (80.1%) against MCF-7 at 50 μg mL^−1^.	[34]
AgNps	*Peltophorum pterocarpum*	Leaf	20–70 nm	Globular	MCF-7	AgNps from *P. pterocarpum* leaf extract showed anticancer activity on MCF-7, with an IC_50_ value of 62 μg mL^−1^.	[48]
AgNps and iron nanoparticles (FeNps)	*Blumea eriantha*	Whole plant	50 nm	Spherical	MCF-7	AgNps from *B. eriantha* whole plant extract induced apoptosis in MCF-7.	[49]
AgNps	*Allium sativum*	Garlic cloves	10–50 nm	Spherical	MCF-7	AgNps from garlic cloves significantly inhibited MCF-7 viability at 100 μg mL^−1^.	[41]
Bimetallic silver-platinum nanoparticles (AgPtNPs)	*Vernonia mespilifolia*	Whole plant	35.5 ± 0.8 nm	Spherical	MCF-7	AgPtNPs from *V. mespilifolia* whole plant extract demonstrated selective cytotoxic potency towards MCF-7.	[50]
AgNps	*Artemisia carvifolia*	Seeds	80 ± 6 nm	Polyhedral	Liver cancer (HepG2) cell lines	AgNps from *A. carvifolia* seed extract showed significant cytotoxicity against HepG2 cell lines, with an IC_50_ value of 2.57 μM. Apoptotic effects were also recorded.	[35]
AgNps	*Cucumis prophetarum*	Leaf	90 nm	Polymorphic (irregularly granulated, ellipsoidal, and highly aggregate)	HepG2	AgNps from *C. prophetarum* leaf extract showed antiproliferative effects on HepG2 cell lines, with an IC_50_ value of 94.2 μg mL^−1^.	[51]
AgNps	*Nepeta deflersiana*	Aerial part	33 nm	Spherical	Human cervical cancer (HeLa) cells	AgNps from *N. deflersiana* aerial parts induced dose-dependent cytotoxicity in HeLa cells and induced apoptosis and necrosis cell death through SubG1 cell cycle arrest.	[45]
AgNps	*Sterculia foetida*	Seeds	6.9 ± 0.2 nm	Spherical	HeLa	AgNps from *S. foetida* seed extract showed antiproliferative (>90%) activity against HeLa cells at a concentration of 16 μg mL^−1^.	[52]
AgNps	*Punica granatum*	Leaves	46.1–61.69 nm	Crystalline	HeLa	AgNps from *P. granatum* leaf extract induced apoptosis in HeLa cells by fragmenting the DNA.	[53]
AgNps	*Azadirachta indica*	Fruit and leaves	14–19 nm	Spherical	Lung cancer cell line (H1975)	AgNps from *A. indica* fruit and leaf extracts were highly toxic against H1975, with IC_50_ values of 62.2 and 91 μg mL^−1^.	[44]
AgNps	*Erythrina indica*	Root	20–118 nm	Spherical	Lung cancer	AgNps from *E. indica* root extract showed cytotoxic effects on lung cancer cell lines.	[54]
AgNps	*Cleome viscosa*	Fruits	20–50 nm	Spherical	Lung cancer (A549)	AgNps from *C. viscosa* fruit extract showed substantial anticancer activities on AS49 cells, with an IC_50_ of 28 μg mL^−1^.	[55]
AgNps	*Dimocarpus longan*	Peel	8–22 nm	Spherical	Human lung cancer (H1299)	AgNps from *D. longan* peel extract showed a strong inhibitory effect on the growth of H1299 cells, associated with their effects on NF-κB, Bcl-2, caspase-3, and survivin.	[56]
AgNps	*Alternanthera sessilis*	Leaves	30–50 nm	Spherical	Human prostrate (PC3) cancer cell line	AgNps from *A. sessilis* leaf extract exerted cytotoxic effects on PC3 cells, possibly via an apoptosis-dependent pathway.	[57]
AgNps	*Dimocarpus longan*	Peel	8–22 nm	Spherical	Prostate cancer (VCaP)	AgNps from *D. longan* peel extract induced a concentration-dependent cytotoxicity against the prostate cancer VCaP cells.	[56]
AgNps	*Carica papaya*	Leaves	10–25 nm	Spherical	Human prostate (DU145)	AgNps from *C. papaya* leaf extract showed anticancer activities via cell cycle arrest and induction of apoptosis.	[58]
AgNps	*Senegalia senegal*	Gum arabic	1–30 nm	Spherical	Colon cancer cell lines (Caco-2 and HT-29)	AgNps from gum arabic demonstrated selected cytotoxicity on colon cancer cell lines.	[42]

## Data Availability

Data will be made available upon reasonable request.

## Supplementary Materials

The following supporting information can be downloaded at https://www.mdpi.com/article/10.3390/plants13202836/s1: Table S1: Main information on global research trends in medicinal plant-synthesized nanoparticles for cancer treatment; Table S2: Foremost 10 productive authors and their citation indices; Table S3: Top 10 highly cited publications.
