# Supplementary material for: Global Research Trends and Recent Advances in Medicinal Plant-Synthesized Nanoparticles for Cancer Treatment
Source: Plants (Basel). 2024 Oct 10;13(20):2836. doi: 10.3390/plants13202836 (PMC11511196; doi:10.3390/plants13202836)
Supplement: Supplementary file 1 [file plants-13-02836-s001.zip › plants-3210238-supplementary.pdf]

Supplementary Table S1. Main information on global research trends in medicinal plant-synthesized nanoparticles for cancer treatment

Data information	Results
Time span	2005:2023
Sources (Journals, Books, etc.)	1168
Documents	4728
Average years from publications	3.29
Average citations per document	23.32
References	284482
**Document content**	
Keywords Plus (ID)	19869
Author's Keywords (DE)	8282
**Authors**	
Authors	16634
Author Appearances	28884
Authors of single-authored documents	98
Authors of multi-authored documents	16536
**Authors collaborations**	
Single-authored documents	119
Documents per Author	0.284
Authors per Document	2.69
Co-Authors per Documents	3.52
Collaboration Index	3.59

Supplementary Table S2. Foremost 10 productive authors and their citation indices.

Authors	H-index	g-index	m-index	Articles	TC	PY start
Steinmetz N	27	40	2.2500000	74	1673	2012
Wang Y	31	55	2.5833333	63	10770	2012
Singh S	18	43	1.3846154	54	6061	2011
Khan M	16	39	2.0000000	52	1585	2016
Zhang X	22	43	1.5714286	50	8380	2010
Wang C	27	44	1.9285714	45	6734	2010
Zhang Y	22	38	1.4666667	44	8698	2009
Li Y	19	38	1.9000000	41	3284	2014
Liu Y	17	31	2.4285714	41	5877	2017
Wang J	17	31	2.1250000	39	5086	2016

Citation indices as at 23 October 2023 (H-index, g-index, m-index); total citation (TC); publication start year (PSY).

Supplementary Table S3. Top 10 highly cited publications

S/N	Article	DOI	TC	TC per year
1	Kuppusamy P, 2016, Saudi Pharm J.	10.1016/j.jsps.2014.11.013	693	86.6
2	Basnet P, 2011, Molecules	10.3390/molecules16064567	560	43.1
3	Yingchoncharoen P, 2016, Pharmacol Rev.	10.1124/pr.115.012070	491	61.4
4	Kasthuri J, 2009, Colloids Surf B Biointerfaces	10.1016/j.colsurfb.2008.09.021	485	32.3
5	Dipankar C, 2012, Colloids Surf B Biointerfaces	10.1016/j.colsurfb.2012.04.006	463	38.6
6	Bonifácio Bv, 2013, Int J Nanomed.	10.2147/IJN.S52634	442	40.2
7	Tomeh Ma, 2019, Int J Mol Sci.	10.3390/ijms20051033	438	87.6
8	Greenwell M, 2015, Int J Pharm L Sci Res.	10.13040/IJPSR.0975-8232.6(10).4103-12 437	437	48.6
9	Zhang M, 2016, Biomaterials.	10.1016/j.biomaterials.2016.06.018	420	52.5
10	Xu L, 2020, Theranostics	10.7150/thno.45413	404	101.0
